# A third of patients with multiligament knee injuries exhibit radiological signs of patella dislocation

**DOI:** 10.1002/jeo2.70228

**Published:** 2025-04-11

**Authors:** Antonio Klasan, Harald Kreuzthaler, Angelika Schwarz, Christian Kammerlander, Thomas Neri, Justin J. Ernat

**Affiliations:** ^1^ AUVA UKH Steiermark Graz Austria; ^2^ Johannes Kepler University Linz Linz Austria; ^3^ Medical University of Graz Graz Austria; ^4^ University Hospital of Saint‐Etienne Saint‐Priest‐en‐Jarez France; ^5^ Laboratory of Human Movement Biology (LIBM EA 7424), University of Lyon‐Jean Monnet Saint Etienne France; ^6^ Department of Orthopedic Surgery University of Utah Health Salt Lake City Utah USA

**Keywords:** cartilage, MPFL, multiligament knee, patella dislocation

## Abstract

**Purpose:**

Multiligament knee injury (MLKI) is considered major trauma. What is currently undescribed is the incidence of concomitant patella dislocation in a setting of MLKI, which is needed for a better understanding of concomitant injuries that are a consequence of the dislocated patella ‐ rupture of the medial patellofemoral ligament (MPFL) and cartilage lesions. The present study aimed to investigate the incidence of patellar dislocation in the MLKI setting.

**Methods:**

Magnetic resonance imaging (MRI) and operative note review of two major trauma centres for 2016–2023 were performed. MLKIs, defined as a disruption of at least two major knee ligaments, are classified as either ACL‐ or PCL‐based or cruciate. All cases had a preoperative MRI and were treated surgically. Patella dislocation on MRI was defined as: (1) dislocated patella, (2) lateralization >2/3 with a bone bruise/cartilage injury, (3) bony or intrasubstance MPFL rupture with or without a bone bruise/cartilage injury. Note was also made on whether the patella was displaceable during surgery. The incidence of MPFL reconstructions was noted via the operative notes.

**Results:**

A total of 364 MKLIs were included. Mean age was 36.0 ± 13.4 years, 131 patients were female (36.0%). Observed incidence of patella instability was 29.7%: disruption of the MPFL was 75 cases (20.6%), lateralization of the patella in combination with bone bruise/cartilage injury in 30 cases (8.2%) as well as one case of a dislocated patella on MRI (0.27%). MPFL reconstruction was performed in 14 cases, and in 2 cases, a repair was performed (4.4%).

**Conclusion:**

The present study demonstrates that the incidence of patella dislocation in the setting of MLKI can be as high as 29.1%. The clinical relevance of currently diagnosing and managing patellar dislocation in the setting of MLKI requires further research.

**Level of Evidence:**

Level III, retrospective study.

**Abbreviations**
ACL: anterior cruciate ligament
LCL: lateral collateral ligament
MCL: medial collateral ligament
MLKI: multiligament knee injury
MPFL: medial patellofemoral ligament
PCL: posterior cruciate ligament
PLC: posterolateral corner

## INTRODUCTION

Multiligament knee injuries (MLKIs) and knee dislocations (KD) are rare but devastating injuries with potentially lifelong consequences [13]. The fundamental principle around MLKI is the disruption of two of the four major knee ligaments, anterior cruciate ligament (ACL), posterior cruciate ligament (PCL), medial collateral ligament (MCL) and lateral collateral ligament (LCL) [17]. Concomitant injuries, as is the case with isolated ligament injuries, can involve meniscus or chondral injuries [12].

The most commonly used classification to describe the injury pattern is the Schenck classification [20]. It was initially used for KD but is essentially used for MLKI and KD. It evolves around the anatomy and the described injury patterns, with the central (I and II), central+medial/lateral (III), complete (IV) or combined with a fracture (V). While the Schenck classification is ligamentously descriptive, it does not reflect any type of chondral or meniscal injury and does not distinguish between PCL‐based and non‐PCL‐based injuries that have been demonstrated to have fundamentally different outcomes [13, 14]. Finally, involvement of the popliteal artery or the peroneal nerve, both not uncommon and with catastrophic outcomes, are also not involved [17].

However, one of the most important aspects of the knee joint is the patellofemoral joint. This ‘second joint’ is largely uncovered within this setting. There are multiple treatment algorithms on how to treat patella dislocation [6], both traumatic and habitual. Still, the patella does not seem to play a major role in current MLKI treatment discussions. Disruption of the patellar tendon has been reported in low‐energy trauma, with recommendations of a two‐stage procedure [4, 22]. MPFL tears have been described to occur in 16.3% of MLKIs [24] or even in up to 59% of MLKIs, but rarely have a secondary clinical instability [1].

The purpose of the present study was to investigate the incidence of radiographic evidence of patellar dislocation in the setting of MLKI. Due to the lack of previous studies, we hypothesized that signs of patella dislocation in the setting of MLKI occur in more than 5% of cases.

## METHODS

This is a retrospective study of operative notes and magnetic resonance imaging (MRI) of patients surgically managed for an MLKI. The study was conducted in two level III trauma centres, AUVA UKH Hospital in Graz, Austria and Department of Orthopedics, University of Utah, Salt Lake City, UT, USA, ethics board AUVA 03/2023 and IRB_00174976. Included were patients managed between 1 January 2016 and 31 December 2023. There were six surgeons in the first centre and four in the second, all experienced in the management of MLKIs. Inclusion criteria were: Grade II or III rupture, of at least two major knee ligaments (ACL, PCL, MCL, LCL/PLC and POL) that underwent surgical management, available preoperative imaging and available intraoperative notes. Injuries were further classified as either cruciate, ACL‐ or PCL‐ based [10, 14]. We put no restrictions on age. Excluded were patients with fracture dislocations.

### Criteria of patella dislocation

**Figure 1:**
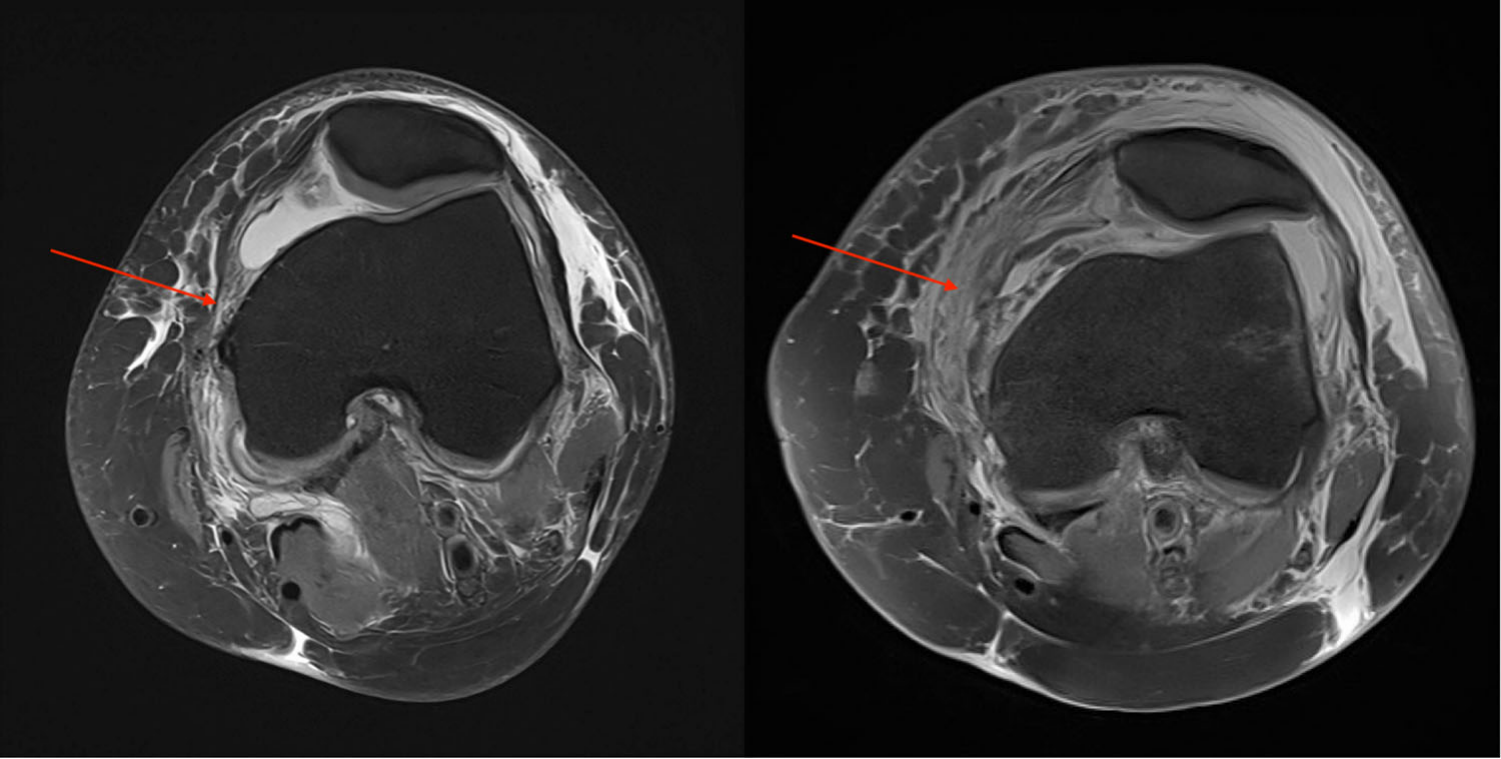
(a, b) MPFL disruption, red arrows. MPFL, medial patellofemoral ligament.

**Figure 2:**
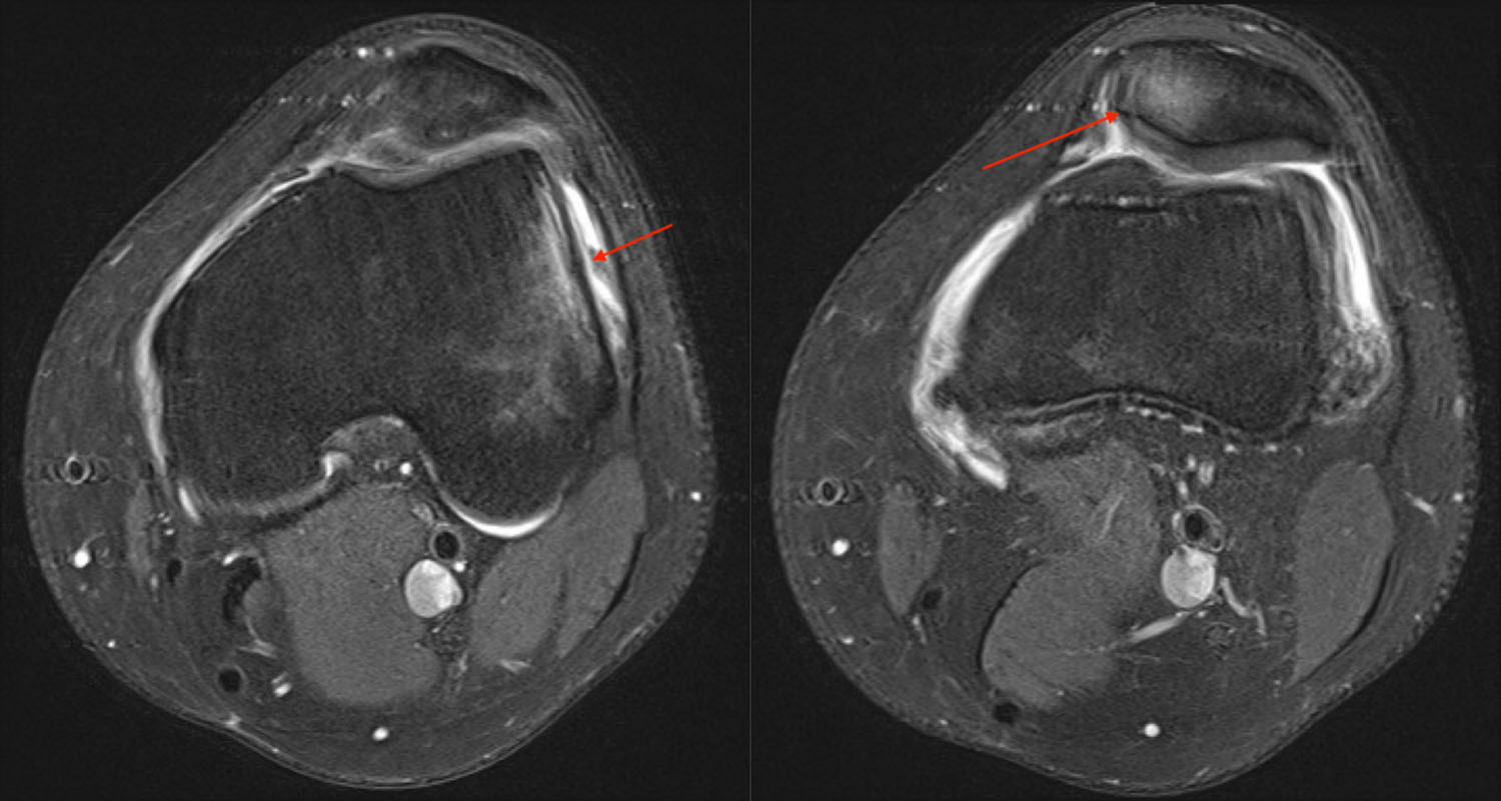
(a, b) Lateral bone bruise of the lateral femoral condyle and medial bone bruise of the patella, red arrows.

**Figure 3:**
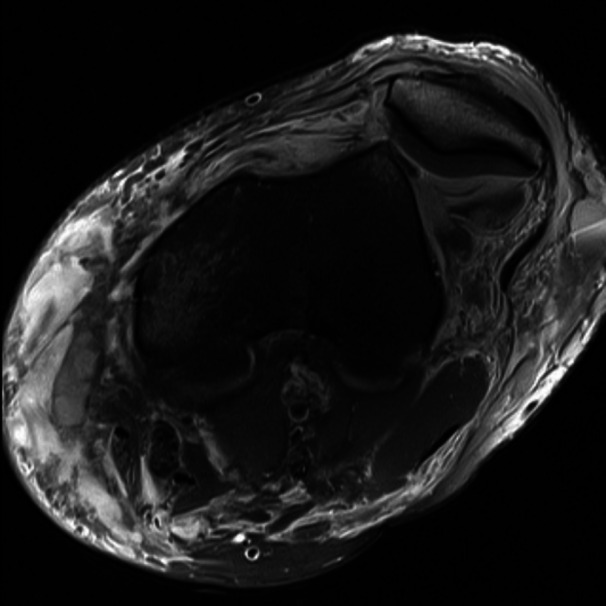
Dislocated patella.

All MRIs were performed within 3 months of the injury, using standardized 3 T MRI protocols [21]. On MRI, the following criteria were followed to diagnose a dislocated patella: [9, 23] disruption of the medial patellofemoral ligament (MPFL) with/without lateralization of patella >2/3 width (Figure 1a,b); bone bruise/cartilage injury of the patella, lateral condyle or both, with/without lateralization of patella >2/3 width (Figure 2); obviously dislocated patella (Figure 3). Intraoperative reports were screened for description of cartilage injuries and cross‐referenced with the MRI findings. A clinically unstable patella during the examination, performed by the surgeon under anaesthesia, was documented. Luxation of the patella in extension and 30° of flexion was investigated.

Finally, cases where an MPFL reconstruction was performed were recorded.

MRIs were evaluated two times by two experienced, fellowship‐trained sports knee surgeons with a minimum of 4 weeks of difference between evaluations. Additionally, two orthopaedic residents performed the same measurements using the same protocol, giving a total of 4 evaluations per case. The consensus was used as the final evaluation in the case of >75% majority consensus. If no consensus could be reached, the case was discussed, and a consensus was obtained. Intra‐ and interobserver consistency of measurements was calculated using the intraclass correlation coefficient (ICC).

### Statistical analysis

Due to a lack of comparative data, a descriptive analysis was performed using the criteria described. A two‐step logistic regression was performed in order to establish the pattern of injury with a higher likelihood of patellar dislocation. In the first step, all four major ligaments and cruciate injuries were analysed with radiographic signs of patellar dislocation as the dependent variable. In the second step, a model was created based on the ligaments with statistical significance at step one. SPSS 29.0.1 (IBM) was used for the analysis. Statistical significance was set at *p* < 0.05. The effect size was reported separately as an odds ratio with a 95% confidence interval and Nagelkerke *R* ^2^.

## RESULTS

A total of 364 MKLIs were included in the study: 186 from the first centre and 178 from the second. A total of 21 patients were excluded due to inconsistent intraoperative reports and 15 patients due to lacking MRI imaging. The mean patient age was 36.0 ± 13.4 years, and 131 patients were female (36.0%). The mean BMI was 29.4 ± 4.4. PCL‐based injury was present in 155 cases (42.6%) and bicruciate injury in 103 cases (28.2%). Meniscal injury was present in 180 cases (49.5%), and cartilage lesion of any area was present in 56 cases (15.4%).

Disruption of the MPFL was observed in 75 cases (20.6%) (Table 1). Lateralization of the patella and bone bruise/cartilage injury was observed in another 30 cases, in addition to the MPFL disruption (8.2%) (Table 1). One case of a dislocated patella on MRI was observed (0.3%). The patellar tendon was ruptured in nine cases (2.5%), and there was one case of quadriceps tendon rupture (0.3%). The incidence of cartilage injury to the patellofemoral joint surfaces was 13.4%. Intraobserver ICC was 0.95, and interobserver ICC was 0.89, highlighting excellent consistency.

**Table 1:** Incidence of MPFL rupture, bone bruise and cartilage injury of the patellofemoral joint, depending on the type of injury.

Type of injury	MPFL disruption	Bone bruise	Cartilage injury
ACL‐based*	71/302 (23.5%)	69/302 (19.7%)	37/302 (12.3%)
PCL‐based	44/155 (28.4%)	34/155 (21.9%)	29/155 (18.7%)
Bicruciate	41/103 (39.8%)	24/103 (23.3%)	18/103 (17.4%)

*Note*: *ACL‐based injuries involve the ACL and collateral ligaments. PCL‐based only involves the PCL and the collateral ligaments. Bicruciate involves both cruciate and collateral ligaments.

Abbreviations: ACL, anterior cruciate ligament; MPFL, medial patellofemoral ligament; PCL, posterior cruciate ligament.

Based on the intraoperative notes, due to a displaceable patella intraoperatively, an MPFL reconstruction was performed in 14 cases (3.8%), and a repair was performed in 2 cases (4.4%). Of those 16 cases, 15 had MRI signs of patella dislocation, which was confirmed intraoperatively. The final patient was the patient with the dislocated patella on MRI.

The injury pattern associated with the highest risk of patella dislocation is a bicruciate injury combined with an MCL injury (Table 2), although *R* ^2^ for the model was 0.156. Younger age was a predictive factor of patella dislocation (*p* = 0.038; odds ratio 0.97, 95% CI: 0.94–1.0). Gender was not a predictive factor (*p* = 0.986).

**Table 2:** Logistic regression analysis of radiologic signs of patella dislocation in combination with other ligaments.

Ligament	*p* value	Odds ratio (95% CI)	*p* value	Odds ratio (95% CI)
ACL	0.005	4.46 (1.56–12.7)		
PCL	0.002	2.28 (1.36–3.81)		
ACL + PCL	<0.001	4.41 (2.59–7.53)	<0.001	4.92 (2.83–8.52)
MCL	0.026	1.99 (1.08–3.62)	0.006	2.45 (1.30–4.65)
PLC	0.702	0.90 (0.53–1.53)		

Abbreviations: ACL, anterior cruciate ligament; CI, confidence interval; MCL, medial collateral ligament; PCL, posterior cruciate ligament; PLC, posterolateral corner.

## DISCUSSION

The most important finding of the present study is a previously undescribed incidence of radiological signs of patella dislocation in the setting of MLKI. The signs of dislocation can be as high as 29.1%. The most common setting is a bicruciate injury involving the MCL, with an odds ratio of 4.92. Younger age is a predictive factor for this injury.

The anteromedial aspect of the knee has received significant attention recently with an increased effort to stabilize an anteromedially unstable knee, involving ACL and superficial medial collateral ligament and posterior oblique ligament (POL) injuries [2, 16]. In a combined valgus and external rotational force, an MCL injury will occur [3]. Adding a valgus force to a knee with an externally rotated tibia in a slightly flexed hip is the most common mechanism of traumatic patella dislocation [7]. The anteromedial complex of the knee extends anteriorly to the patella via the MPFL [11, 18]. As the present study demonstrates, a medial‐sided injury more often involves the MPFL and the patella, as perhaps previously believed.

The traditional classification of MLKIs is the classification proposed by Schenck in 1994 [20]. It pertains primarily to the dislocated knee but has been expanded to be used interchangeably with MLKI in multiple studies. A recent consensus study has determined the need to create a new classification system [17], and such attempts have been made previously [8]. It remains to be seen if the new proposals will incorporate the patellofemoral joint and the dislocated patella.

Patella dislocation in this setting has been largely uninvestigated. Allen et al. investigated 21 patients after an MLKI with radiological signs of an MPFL tear [1]. At 3.6 years, one of those patients complained about patellofemoral instability symptoms. Zheng et al. investigated 219 MRIs after acute isolated patella dislocations or MLKIs [24]. They found MPFL tears to be relatively uncommon in MLKIs, up to 18%.

A somewhat better‐investigated issue is the disruption of the extensor mechanism in an MLKI setting, either the quadriceps or the patellar tendon. It complicates and delays treatment significantly. Chun et al. investigated a two‐hospital database and found the incidence of patellar tendon rupture within the multiligament setting to be 13% [4]. The authors recommend a staged approach, which has been reported in the literature on a case‐series basis as successful [22]. A recent consensus paper pointed out the severity of the injury involving the extensor mechanism, also recommending adding the extensor mechanism to a yet‐to‐be‐created new classification of extensor mechanism injuries [15]. While patellar instability and dislocation is a different pathology than extensor mechanism injury, we feel that further study of the extensor mechanism and its stabilizing components is critical to understanding the complexities of MLKIs and knee dislocations.

Patella dislocation is a complex issue. However, the treatment algorithm in the acute setting is fairly straightforward. Without chondral damage and severe instability, non‐surgical management should be pursued [6]. Similarly, a mono‐ligament knee injury, typically ACL without concomitant injuries, can be reasonably managed non‐operatively in selected patients [19]. In a multiligament setting, all issues become more complex, the outcomes worsen [13], and the general consensus is surgical management [17]. It is unclear from the literature and from this study if patella dislocation in the MLKI setting has long‐term clinical relevance. It is also unclear whether the MPFL should be repaired, augmented or reconstructed in this setting, due to the complexity of other co‐factors. The first step, however, is considering the patellofemoral joint in the treatment algorithms of MLKI, as it keeps being forgotten in the setting of osteoarthritis [5].

This study has some limitations. The retrospective design does not allow systemic screening and examination documentation under each patella's anaesthesia. Not all MPFL disruptions automatically mean the patella was dislocated or even subluxated; however, lack of any MPFL injury means the patella remained in situ during the injury, or the trauma bypasses this component of the knee joint. The clinical relevance of the MPFL reconstructions performed in the study is unclear, but the primary purpose was to investigate the incidence of dislocation, not to compare outcomes. As the MPFL reconstruction was performed at the surgeons' discretion, this aspect of the study should be interpreted with caution.

## CONCLUSION

The present study demonstrates that the previously undescribed incidence of radiological signs of patella dislocation in the setting of MLKI can be as high as 29.1%, most commonly associated with an ACL, PCL or MCL injury. The clinical relevance of currently diagnosing and managing patellar dislocation in the setting of MLKI requires further research.

## AUTHOR CONTRIBUTIONS

Antonio Klasan, Justin J. Ernat and Thomas Neri conceived the study. Antonio Klasan, Harald Kreuzthaler and Justin J. Ernat performed the evaluation. Antonio Klasan and Angelika Schwarz performed the analysis. Antonio Klasan and Angelika Schwarz wrote the first draft. Christian Kammerlander, Thomas Neri and Justin J. Ernat revised it. All authors have read and approved the manuscript.

## CONFLICT OF INTEREST STATEMENT

Antonio Klasanis is an associate editor for the *Journal of Knee Surgery* and an editorial board member of *Archives of Orthopaedic and Trauma Surgery* and *Knee Surgery, Sports Traumatology, Arthroscopy*. He has been paid to present by Arthrex and Implantcast. Justin J. Ernat is a consultant for DePuy Synthes. The remaining authors declare no conflicts of interest.

## ETHICS STATEMENT

Ethics board AUVA 03/2023 and IRB_00174976. As per ethics board approvals, no consent was required for a retrospective study.

## Data Availability

Data are available upon request.
